# Do Ultrasound Measures of Bladder Neck Location and Movement During Pelvic Floor Contraction or Straining Relate to Bladder Neck Support During Voiding?

**DOI:** 10.1002/nau.70307

**Published:** 2026-05-14

**Authors:** Bernadette Dellar, Eric Chung, Paul W. Hodges

**Affiliations:** ^1^ Centre for Innovation in Pain and Health Research, Faculty of Health, Medicine and Behavioural Sciences The University of Queensland Brisbane Queensland Australia

**Keywords:** diagnostic techniques, lower urinary tract symptoms, pelvic floor muscle contraction, straining, ultrasound, urination

## Abstract

**Purpose:**

Poor support of the bladder neck is common in voiding dysfunction but remains difficult to assess. Transperineal ultrasound imaging during voiding provides reliable measures but is not yet implemented in practice. This study aimed to investigate whether conventional measures of bladder neck position during straining and pelvic floor muscle contraction correlate with support during voiding, potentially serving as surrogate measures. A secondary objective was to compare the measures between nulliparous and parous participants.

**Materials and Methods:**

Thirty asymptomatic women underwent transperineal ultrasound to measure bladder neck position during voiding, maximal straining, and maximal pelvic floor muscle contraction. Measurements included the pubourethral angle and the distance between the symphysis and bladder neck, recorded at rest, start and end of voiding, and during maximal strain and pelvic floor muscle contraction. Correlations were calculated between the end position and changes in bladder neck position during voiding and during strain or contraction.

**Results and Conclusions:**

No significant correlations were found between the *changes* during voiding and the other tasks. Moderate to strong correlation were observed between bladder neck position at the end of voiding and the other tasks. There was no significant differences between nulliparous and parous participants. The findings suggest that the bladder neck displacement during voiding cannot be inferred from the movements during strain or pelvic floor muscle contraction. Although there is correlation between position at the end of each task, this does not reflect the dynamic change in voiding, which is likely crucial for clinical assessment and understanding of voiding dysfunction.

## 1 Introduction

Modified support of the pelvic structures, including that of bladder neck, constitutes an important feature in female pelvic health. Although its role in prolapse and incontinence is well‐studied, the role in voiding and voiding dysfunction (VD) has received less attention. VD refers to an abnormally slow and/or incomplete micturition [1] and often occurs alongside other disorders [2], with 62% of women with pelvic floor dysfunction reporting VD symptoms [3]. Support of pelvic structures relies on the coordination of the levator ani muscles as well as passive contributions from ligaments and fascia [4]. Structural damage or dysfunction may arise from a variety of factors, including genetics [3], abnormalities in collagen composition, hormonal fluctuations, and obstetric or pelvic trauma [5]. Assessment of bladder neck support can provide important information regarding the underlying mechanisms explaining dysfunction of support and this might present differently in different tasks.

Assessment of the pelvic region with ultrasound imaging is well established during different maneuvers to assess different aspects of support and muscle function [6]. The two most common tasks assessed with ultrasound are straining and pelvic floor muscle (PFM) contraction. Straining is used to assess the capacity of passive and active pelvic floor support when intra‐abdominal pressure (IAP) is elevated [7] and is assumed to reflect vaginal wall/bladder neck support in functional activities, prolapse and incontinence [8]. Contraction of PFM evaluates the capacity of the PFM to contract and shorten [9]. Both maneuvers, are performed with an empty bladder and the bladder neck closed. Whether they provide information of bladder neck support during micturition is unclear.

The bladder neck and urethra are support during voiding [10, 11]. Abnormal bladder neck support can interfere with voiding and must be differentiated from other causes of VD [12, 13]. The assessment of bladder neck position during voiding has not traditionally been part of standard practice. It is plausible that the ability to elevate the bladder neck during PFM contraction and during straining could be sufficiently related to support during voiding to provide a surrogate measure. This relationship has not yet been systematically investigated.

A new method has been developed for detailed evaluation of voiding with transperineal ultrasound imaging. Voiding Sonography (VS) enables assessment of micturition [14] including the expulsion of urine through the urethra via the bladder neck. This study aimed to assess whether bladder neck support during voiding is related to bladder neck position and movement during straining or PFM contraction in women without pelvic floor dysfunction symptoms. A secondary aim was to compare these measures between nulliparous and parous women.

## 2 Materials and Methods

### 2.1 Participants

Participants were included if they were aged between 18 and 55 years and had no symptoms of urogenital dysfunction defined by a score of < 12 on the International Consultation on Incontinence Questionnaire – Urinary Incontinence Short Form (ICIQ‐UI SF) and < 6 on the ICIQ‐VS (Vaginal Symptoms). They were also excluded if they had a history of other pelvic floor conditions, any major neurological condition or diabetes. Women were eligible if they were nulliparous or parous. Participants had to be able to void completely, with post void residue (PVR) bladder volume of less than 50 mL. Participants were also involved in a second session for a separate study [15]. The Institutional Human Research Ethics Committee (2021/HE000137) approved the study. Participants provided written and informed consent.

### 2.2 Ultrasound Imaging

VS was performed by the sonographer with 20 years of experience including development of this technique. A General Electric (GE) ultrasound machine (E8 model) with a C1‐5, 1–5 MHz frequency, 2D convex footprint with a 70‐degrees wide field of view transducer was used. The ultrasound machine's in‐built measurement requirements include distances in mm, measurement of angles via a caliper tool and continuous DICOM video recording throughout the entire void process (minimum duration of 90 s).

### 2.3 Voiding Sonography Technique

**FIGURE 1:**
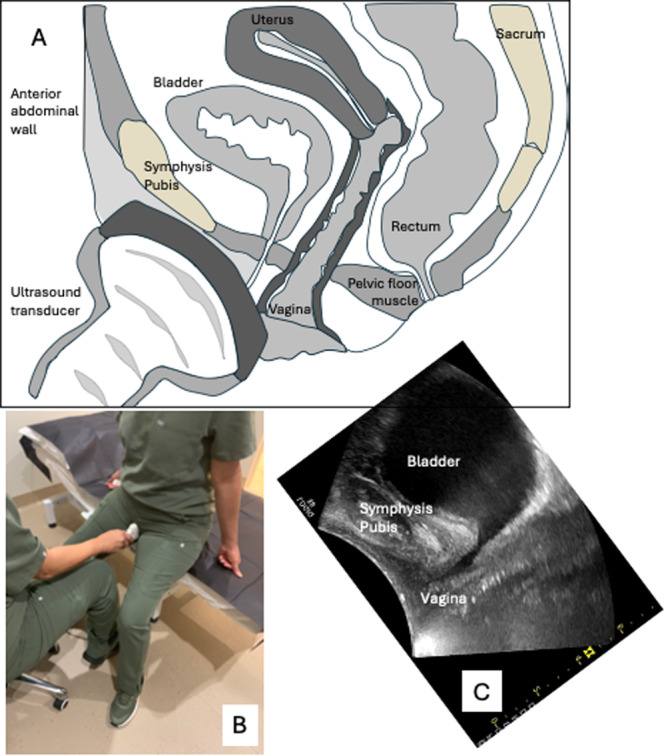
Participant and ultrasound transducer position during voiding and other tasks. (A) Sagittal plane line diagram showing the ultrasound transducer position relative to anatomical structures. The symphysis pubis serves as the fixed landmark. (B) Demonstration of transducer placement on the perineum of a clothed subject. Note that during the experiment the participant was unclothed and positioned on a commode chair to enable capture of urine in the uroflow. The covered ultrasound transducer is positioned in direct contact with the mons pubis. (C) Example ultrasound image showing the pelvic structures with the same orientation as the line drawing in panel A.

A comprehensive technical overview of Voiding Sonography was provided by Dellar et al. [14] and reliability of the procedure has been demonstrated [15]. The procedure was conducted with participants in a seated position, with a uroflowmetry device positioned beneath the commode to measure urine flow parameters [14]. Figure 1 illustrates the transducer positon, which allows unobstructed urine flow during micturition. The same transducer placement is maintained during PFM contraction and straining tasks. The entire symphysis pubis (SP) fibrocartilage disc is visualized [14].

### 2.4 Procedure

Participants followed a bladder filling protocol that involved consumption of 250 mL of a non‐diuretic fluid hourly for 3 h (750 mL total) and arrived for testing with a full bladder. Bladder fullness volume was confirmed via transabdominal ultrasound in the supine position when the urge to void was reported and measured using standard calculations [16]. Participants sat on a commode. Using aseptic technique, the sonographer positioned the transducer in the mid‐sagittal plane, with one edge placed over the mons pubis (Figure 1). The length of the SP defined as the distance between the superior and inferior margin along the mid‐pubic line (MPL) [17] was measured. As the only fixed landmark (Figure 1), the SP measurement ensured that all subsequent measures were obtained from a consistent reference plane. Participants were instructed to void as normally as possible. The sonographer continuously monitored the procedure to assess the completeness of voiding, confirmed by visualization of an empty bladder and absence of residual urine on ultrasound. Participants rested for several minutes before proceeding to the next task. Participants were instructed to relax their abdominal and pelvic floor muscles, take two deep breaths, and perform a 5 s maximal strain maneuver. This task involved increasing intra‐abdominal pressure with a closed mouth and forcing the abdominopelvic contents caudally while maintaining relaxation of the PFM to allow the pelvic floor to descend [7]. After 1–2 min rest, participants were instructed to contract their PFM maximally, using the mental image of the bladder/urethra moving upward and forward in a ventral cephalad direction [6]. Participants were requested to perform the task with maximum effort using strong verbal encouragement, hold the contraction for 5 s and then relax.

### 2.5 Data Analysis

**FIGURE 2:**
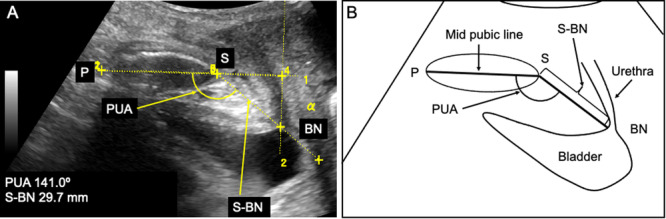
Ultrasound measures. (A) Ultrasound image at maximum straining and measures. (B) Line drawing of the ultrasound image. The mid pubic line is a central longitudinal line from the superior border (P) to the inferior border (S) of the symphysis pubis; (i) pubourethral angle (PUA) is the angle between line PS; and symphysis to bladder neck (S‐BN) is measured along a line from inferior border of the PS and bladder neck.

Recorded ultrasound videos were analyzed post‐procedure. The sonographer selected the images for measurements. Measurements were performed and saved on the ultrasound machine. Measures were: pubourethral angle (PUA)—angle between the MPL to the anterior bladder neck junction (ABNJ), and anterior bladder neck distance to symphysis distance (S‐BN)—distance between the inferior margin of the MPL to the ABNJ [14] (Figure 2).

**FIGURE 3:**
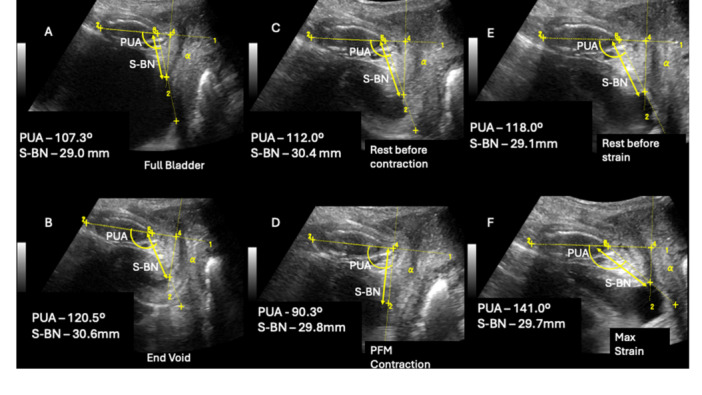
Example of calculation of the change in measures of pubourethral angle (PUA) and symphysis to bladder neck distance (S‐BN). Measures are shown at; (A) Full bladder; (B) End‐of‐void; (C) Rest before contraction; (D) Maximum PFM contraction; (E) Rest before strain; and (F) Maximum strain. Change in PUA calculations for: voiding – (End void‐full bladder), 120.5°−107.3° = 13.2°; contraction of PFM – (contraction‐rest before contraction), 90.1°−112.0° = −21.9°; straining – (strain‐rest before strain), 141.0°−118° = 23°; and calculation for change in S‐BN: voiding – (end void‐full bladder), 30.6mm–29.0 mm =1.6 mm; contraction of PFM – (contraction‐rest before contraction), 29.9mm–30.4 mm = −0.5 mm; strain – (strain‐rest before strain), 29.7mm–29.1 mm =0.6 mm.

Variables calculated for voiding were the PUA and S‐BN at end of void; and the change in PUA and S‐BN from start (with full bladder) to end of void. Positive values for the change in PUA indicate downward motion of the bladder neck whereas negative values indicate bladder neck elevation. Positive values for S‐BN indicate an increase in distance and a negative value indicates reduction in distance. Slight descent of the bladder neck is expected during voiding, and both excessive elevation and descent may demonstrate pathology. Calculations are shown in Figure 3A–F.

Variables calculated for straining and contraction were the PUA and S‐BN position at the maximum of the task, and the change in PUA and S‐BN from rest to maximum of the task. Strain would be expected to be associated with small positive change, and contraction would be expected to induce a large negative change. Maximum mobility was calculated by subtracting the PUA and S‐BN at maximum PFM contraction from that in maximum strain [13].

### 2.6 Statistical Analysis

Pearson's correlation was used to assess the relationship between measures made in voiding and either strain or PFM contraction. Separate analyses were used to evaluate measures made and the end/maximum of each task and the change from start/rest to end of each task. The correlation between change in PUA and S‐BN measures during voiding and the maximum mobility measure was also calculated. T‐test and Box and Whisker plots were used to compare differences between the nulliparous and parous women.

## 3 Results

Thirty‐five asymptomatic women volunteered. Two participants (participants no.31 and no.35) were not able to void with the transducer on their perineum and one participant (participant no.6) did not perform the void due to other medical issues not related to the study. Data for 32 participants were available for descriptive statistics of measures made during the voiding phase of micturition. As data were missing for resting conditions for two participants (ID no.5 and 28) for the strain and PFM contraction maneuvers, data for 30 participants were available for comparison between task. Participant demographic data are presented in Table 1, and individual ultrasound measures at task and corresponding calculated change measures are provided in Supporting Information S1: Tables 1–3.

**TABLE 1:** Participant demographics (*n* = 32).

Parameter		Median	Range
Age (years)		34	(18–53)
BMI (kg/m^2^)		22.4	(18–35)
Parity		19 nulliparous	
		13 parous	
ICIQ – UI – SF (count)		3	(0–10)
ICIQ – VS vaginal symptoms (count)		1	(0–6)
ICIQ – VS bothersome score (count)		0	(0–17)

### 3.1 Ultrasound Measures During Void, Contraction and Straining

During PFM contraction, the change in PUA was negative for all except one participant, which infers elevation of the bladder neck, and 23/30 (77%) had a negative change for S‐BN (indicating reduction in distance). During straining, the change in PUA was positive (indicating bladder neck descent) for 30/30 (100%), and S‐BN was positive (indicating increased distance) for 16/30 (53%) of participants. In contrast to the consistent changes observed for PUA during PFM contraction and straining, there was substantial inter‐participant variation in the change measures during void. During voiding, the change in PUA and S‐BN was negative for 9/30 (30%) and 10/30 (33%) of participants, respectively. Supporting Information S1: Table 1 shows the individual participants’ measures for the position at full bladder and the end of each task. The mean (SD) PUA for end void, contract and strain was 125.3(18.4)°, 92.7(17.1)° and 137.4(20)°, respectively (Table 2). The mean S‐BN for end void, maximum contraction and maximum strain were 27.1(4)mm, 25.6(4.5)mm and 27.5(3.7)mm, respectively (Table 2).

**TABLE 2:** Ultrasound measures of PUA and S‐BN during micturition, PFM contraction and strain.

Ultrasound measures during micturition			
Micturition (*n* = 32)		Mean/median (SD)	Min – max (Range)
PUA (degrees)	Full bladder‐start of void	120.3/118.7 (17.8)	79.3 – 154.7 (75.4)
	End void	125.3/125.1 (18.4)	81.2 – 155.3 (74.1)
	Voiding movement^a^	61/4.1 (17.9)	67.6 – 23.9 (17.9)
S‐BN (mm)	At full bladder	26/26 (3.3)	16.6 – 32.1 (15.5)
	End void	27.1/27.4 (4)	17.4 – 34.7 (17.3)
	Voiding movement^a^	11/7.8 (3.1)	7.8 – 5.3 (3.1)

**Ultrasound measures during PFM contraction and strain**			
**PFM maneuver (*n*** = **30)**		**Mean/median (SD)**	**Min – max (Range)**
PUA (degrees)	Rest before strain	115.3/114.2 (17.1)	65 – 146.2 (81.2)
	Maximum strain	137.4/139.8 (20)	77.6 – 169.1 (91.5)
	Rest before contraction	121.9/124.8 (18.6)	67.7 – 163.4 (95.7)
	Maximum contraction	92.7/90 (17.1)	62.5 – 136.7 (74.2)
	Max mobility^b^	44.7/41.5(18.1)	14.1 – 74.3 (60.2)
S‐BN (mm)	Rest before strain	27.4/27.1 (3.6)	20.4 – 33.3 (12.9)
	Maximum strain	27.5/28.1 (3.7)	21.2 – 33.7 (12.5)
	Rest before contraction	27.2/27.4 (3.7)	18.1 – 34.7 (16.6)
	Maximum contraction	25.6/26.1 (4.5)	17.3 – 34.6 (17.3)
	Max mobility^b^	1.9/1.7 (4.1)	6.1 – 9.7 (4.1)

a Voiding movement calculation = end void‐full bladder.

b Max mobility (PFM) calculation = max strain‐max contraction.

### 3.2 Correlations Between Measures Made at End of Void Versus Maximum Strain and Contraction

**FIGURE 4:**
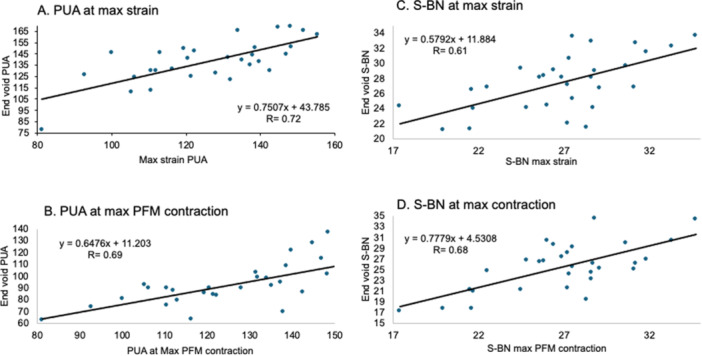
Correlation pubourethral angle (PUA) and symphysis to bladder neck (S‐BN) between end void and pelvic floor muscle (PFM) contraction or strain. (A) PUA at maximum strain versus End‐of‐void; (B) PUA at maximum PFM contraction versus End void; (C) S‐BN at maximum strain versus End‐of‐void; and (D) S‐BN at maximum PFM contraction versus End‐of‐void.

There were strong correlations between PUA at end void and at maximum strain (*r* = 0.72; Figure 4A), PUA at end void and maximum PFM contraction (*r* = 0.69; Figure 4B), and S‐BN at end void and PFM maximum contraction (*r* = 0.68; Figure 4D). The correlation between S‐BN at end void and PFM maximum strain was moderate (*r* = 0.61; Figure 4C).

### 3.3 Correlation Between Measures for Change During Void Versus Change During Maximum Strain and Contraction

**FIGURE 5:**
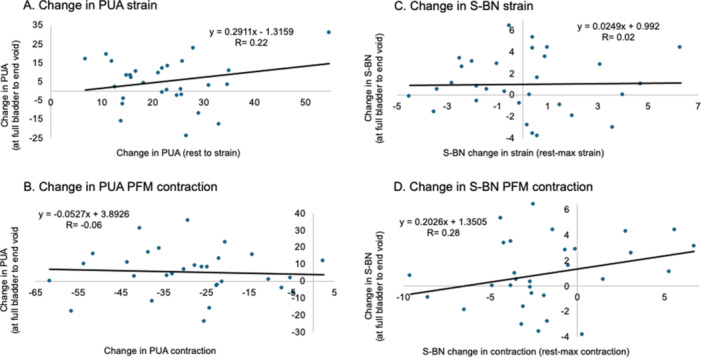
Correlation pubourethral angle (PUA) and symphysis to bladder neck distance (S‐BN) between change in voiding and change in pelvic floor muscle (PFM) contraction or strain. (A) PUA change in strain versus change in voiding; (B) PUA change in PFM contraction versus voiding; (C) S‐BN in strain versus change in voiding; (D) S‐BN change in PFM contraction versus change in voiding.

The mean (SD) change in PUA for voiding, contraction, straining and maximum mobility were 5.4 (5.6)°, −28.8 (−26.9)°, 23.1 (10.2)° and 51.9 (47.4)°, respectively (Table 3). The mean (SD) change in S‐BN for voiding, contraction straining and maximum mobility were 1 (0.6)mm, −1.8 (−2.6)mm, 0.1 (0.3)mm and 1.8 (2)mm respectively (Table 3). In contrast to the static end void and maximum measures, *changes* in both PUA and S‐BN measures demonstrated poor or weak correlations between voiding and strain (S‐BN: *r* = 0.02; Figure 5C; PUA: *r* = 0.22; Figure 5A); and between voiding and PFM contraction (S‐BN: *r* = 0.28; Figure 5D). The correlation between change in voiding and rest to maximum contraction for PUA (*r* = −0.06; Figure 5B) was weak and not significant.

**TABLE 3:** Measures of change with each task.

		Mean/Median (SD)	Min – Max (Range)
**Change in PUA (degrees)**	Voiding (*n* = 32)	**5.4**/5.6 (13.4)	–23.9 – 35.5 (59.4)
	Straining (*n* = 30)	23.1/22.5 (10.2)	6.8 – 54.7 (47.9)
	Contraction of PFM (*n* = 30)	–28.8/–26.9 (15.8)	–61.7 – 2.4 (64.1)
**Change in S‐BN (mm)**	Voiding (*n* = 32)	**1**/0.6 (2.7)	–‐3.8 – 6.4 (10.2)
	Straining (*n* = 30)	0.1/0.3 (2.5)	–4.5 – 6.3 (10.8)
	Contraction of PFM (*n* = 30)	–1.8/‐2.6 (3.8)	–9.6 – 6.7 (16.3)
**Maximum mobility (strain minus PFM contraction)**	PUA (degrees) (*n* = 30)	**51.9**/47.4 (20.5)	17.8 – 95.3 (77.5)
	S‐BN (mm) (*n* = 30)	**1.8**/2 (5)	–8.7 – 10.1 (18.8)

### 3.4 Correlation Between Measures for Change During Void and Maximum Mobility

**FIGURE 6:**
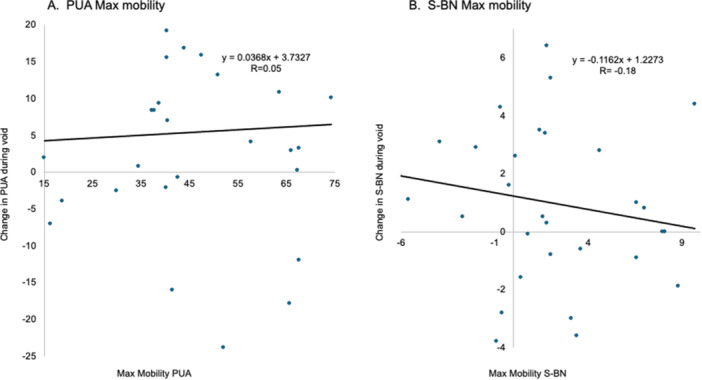
Correlation pubourethral angle (PUA) and symphysis to bladder neck distance (S‐BN) between voiding and maximum mobility. (A) PUA at max mobility versus during voiding; (B) S‐BN at max mobility versus during voiding.

There was a weak positive correlation between change during voiding and maximum mobility for PUA (*r* = 0.05; Figure 6A). Correlations for maximum mobility for S‐BN and change in voiding were weak and negative (*r* = −0.18; Figure 6B).

### 3.5 Comparison Between Nulliparous Versus Parous Participants

The participant group included 12 women who were parous (median [range] age: 43 (18–53); BMI: 24 (18–35); two had 1 delivery and ten had 2–4 deliveries) and 18 who were nulliparous (age: 30 (18–47); BMI: 24 (18–35)). The *change in S‐BN* during voiding was greater in the nulliparous (2 {SD = 2.2}mm) than parous (−0.5 {2.8}mm) group (*p* = 0.014; Table 4). There were no significant differences between groups for the change in PUA during voiding (*p* = 0.136), strain (*p* = 0.829) or PFM contraction (*p* = 0.211); or for S‐BN strain (*p* = 0.835) or PFM contraction (*p* = 0.837). End measures of PUA were not different between groups for void (*p* = 0.954), and strain (*p* = 0.791) but were less for nulliparous (87.7 {12.8}°) than parous (100.6 {20.9}°) participants for PFM contraction (*p* = 0.046; Table 4). There were no significant differences between groups for S‐BN at end void (*p* = 0.487), maximum strain (*p* = 0.803) and maximum PFM contraction (*p* = 0.548).

**TABLE 4:** Comparison between nulliparous and parous women, end void, maximum activity and change variables (*n* = 30).

Variable	Nulliparous (*n* = 18)	Parous (*n* = 12)	p‐value
PUA end void (°)	125.9 (19)	126.3 (18.7)	0.954
PUA max strain (°)	137.7 (22.7)	139.6 (13.8)	0.791
*PUA max contraction (°)*	*87.7 (12.8)*	*100.6 (20.9)*	***0.046***
S‐BN end void (mm)	27.3 (3.9)	26.2 (4.1)	0.487
S‐BN max strain (mm)	27.6 (3.7)	27.2 (3.9)	0.803
S‐BN max contraction (mm)	25.8 (4.1)	24.8 (5.1)	0.548
Change in PUA voiding (°)	8.4 (11.6)	0.9 (15.2)	0.136
Change in PUA strain (°)	23.4 (11.9)	22.6 (7.4)	0.829
Change in PUA contraction (°)	–31.8 (15.6)	–24.3 (15.7)	0.211
*Change in S‐BN voiding (mm)*	*2 (2.2)*	–*0.5 (2.8)*	***0.014***
Change in S‐BN strain (mm)	0 (2.8)	0.2 (2.3)	0.835
Change in S‐BN contraction (mm)	–1.6 (4)	1.9 (3.6)	0.837
Maximum mobility for PUA (°)	49.9 (17.3)	39.1 (16.7)	0.099
Maximum mobility for S‐BN (mm)	1.7 (4.2)	2.4 (4.1)	0.668

## 4 Discussion

The results of this study revealed a relationship between static measures of PUA and S‐BN at end‐void and those made at maximum PFM contraction and strain, but no relationship between the change in these measures during voiding and their change during PFM contraction and straining. Although measures of change and static measures at the end of the task may be useful for assessment of pelvic floor function, distinguishing the mechanisms behind VD is likely to require evaluation of the change during voiding. The lack of relationship between the dynamic change measures in these tasks suggests that measures made during PFM contraction and strain cannot be used as a surrogate for VS.

### 4.1 Changes During Void, Strain and Contraction

Previous work has used fluoroscopy to measure bladder and urethral movements during micturition [18]. These studies identified three key regions involved in voluntary and involuntary micturition: (1) bladder base, (2) urethrovesical segment (bladder neck/proximal urethra), and (3) the voluntary mid‐urethra [19]. Normal voiding begins with flattening and slight descent of the bladder base, increased angle (PUA) and distance (S‐BN) relative to the SP, prior to initiation of micturition. These initial positions reflect the status of the pelvic floor support, IAP or underlying structural dysfunction. Relaxation of the pubococcygeus muscles precedes voiding, leading to bladder neck opening and urethrovesical shortening. As voiding progresses, the bladder base elevates and the segment elongates, with final stages showing a raised bladder and lengthened urethrovesical segment [10]. In women with VD measures made with a transrectal placement of the ultrasound transducer during voiding have shown various difference in pelvic floor control [20]. These include flattening of the posterior urethrovesical angle (PUVA) to 180° during voiding [20] which measures a similar property to our measure of PUA, but from the posterior aspect of the urethrovesicle junction.

The VS measures reported here enabled quantification of the initial support and the change in position that occurs throughout micturition. As an indication of the dynamic support of the bladder neck during voiding, measures of change in angle (PUA) or distance (S‐BN) could provide useful clinical information to discriminate causes of VD. For instance, absence of a change in angle or distance could imply insufficient relaxation/contraction of PFM or excessive passive support preventing the descent and elevation [21]. Greater than expected increase in angle and distance could imply excessive relaxation or inadequate passive support for the bladder base [10] or excessive intra‐abdominal pressure (IAP). Excessive descent or elevation could infer too little or too much support, respectively, and would be likely to compromise the mechanics of voiding. Interpretation of these features requires measurement of the position at the start and end of voiding rather than static end void measures.

Our data show that most of our asymptomatic participants had net descent of bladder neck during micturition, whereas one third had elevation. These finding highlights that although the bladder neck is expected to transition through small descent and elevation during micturition, the net change from the start of void is commonly slight net descent. Participants with bladder neck descent might have more relaxation of the PFM, greater IAP or less passive support. Those with elevation might have greater contraction of PFM, less relaxation and lower IAP. This finding highlights the necessity to investigate VS in asymptomatic and symptomatic women to investigate the normal values for bladder neck motion, the interpretation of variation of measures in asymptomatic women.

Measures of change in bladder neck measures during straining and PFM contraction were not correlated with that during voiding, which indicates they cannot be used as surrogate measures. Notably, measures in these tasks were relatively consistent for all women (elevation with contraction and descent with strain), and this did not reflect the variation in change measures with voiding. Regardless, these measures do provide useful interpretation the capacity of PFM to support the bladder for assessment of stress urinary incontinence and pelvic organ prolapse [22, 23].

### 4.2 Measures of PUA and S‐BN at End of Voiding, and Maximum Contraction and Strain

The relationship between static measures of PUA and S‐BN made at the end of each task suggests these variables tend to vary together; however, their clinical relevance requires further investigation. Similar to dynamic measures, end‐of‐void positions reflect the combined influenced by PFM activity, IAP, and passive structural support. Single timepoint measurements cannot indicate whether these parameters changed during task or remain static. For instance, an end‐void value may reflect a gradual shift or a stable position maintained throughout voiding. Such measures fail to capture the diverse dynamic patterns observed across participants.

Although maximum strain is used to assess the PFM support in POP, it does not indicate if the descent occurred during the task or was present to begin with. Change measures could provide insight into whether the descent is caused by poor passive support, elevated IAP, or lower PFM tone. Pregazzi et al [24], Nyhus et al [25] and Mouritsen et al [13], suggest that poor support is indicated by strain angle > 120°, however, due to difference in transducer position, direct comparison to our PUA data is difficult.

Measures recorded at end PFM contraction can be difficult to interpret. Although a larger PUA and S‐BN might imply that the PFM are contracted, it does not inform whether muscle contracted during the task or was contracted to begin with. Mouritsen et al. [13] reported that poor support occurs when contraction angle < 90°, but the angle can be affected by transducer position, thereby making a direct comparisons to our PUA value difficult.

### 4.3 Comparison Between Asymptomatic Nulliparous and Parous Women

There were minimal differences between nulliparous and parous asymptomatic women across the measured outcomes. Although nulliparous participants demonstrated a greater change in S‐BN during voiding and lower PUA during PFM contraction, there was no differences in other variables. The overall lack of consistent differences suggests parity has limited influence on pelvic floor or bladder neck dynamics when women are asymptomatic of pelvic floor dysfunction. We cannot exclude that the lack of difference might also be related to the insufficient power for this analysis.

### 4.4 Clinical Implications

The poor correlation between conventional bladder neck measures during strain, PFM contraction, and voiding highlights the value of adding VS to assessment of VD. Unlike urodynamics and EMG, VS offers real‐time, non‐invasive imaging of both function and anatomy, enabling visualization of bladder neck position and movement. This makes it a promising complementary tool for evaluating voiding dynamics and distinguishing functional for structural abnormalities.

Before clinical implementation, studies are needed in asymptomatic women to establish normative data, and in populations with various types of VD. Additional VS derived metrics, such as urethral opening and timing specific measurements during voiding should be explored.

## 5 Limitations

The study has several limitations. First, assessments were limited to two‐dimensional mid‐sagittal view, allowing visualization of midline structures but would miss pathology outside this field. Second, the technique required transducer placement on the mons pubis that is potentially intrusive and embarrassing for some participants. Two participants were unable to void, and previous work shows that VS might slightly alter voiding parameters (e.g., void time and peak flow [14]).

## 6 Conclusion

Dynamic changes in PUA and S‐BN during voiding are not reflected by end‐of‐void measures and therefore cannot act as surrogate markers. Voiding sonography provides valuable real‐time assessment of coordination between PFM activity and passive support structures, supporting its role as a complementary diagnostic tool. Pelvic floor and bladder neck dynamics were similar between nulliparous and parous asymptomatic women, suggesting limited influence of parity alone in women who are symptomatic.

## Author Contributions

Bernadette Dellar and Paul W. Hodges designed the study. Bernadette Dellar performed the ultrasound measuremets and clinical evaluations. Bernadette Dellar and Paul W. Hodges analyzed and interpreted the data. All authors (Bernadette Dellar, Eric Chung, Paul W. Hodges) drafted the manuscript. All authors (Bernadette Dellar, Eric Chung, Paul W. Hodges) contributed to revising the article critically for important intellectual content and approved the final version to be published.

## Ethics Statement

The study protocol was approved by the University of Queensland Human Research Ethics Committee. Decision no. 2021/HE000137 Date: 14 December 2021. All analysis performed involving human participants was in accordance with the 1964 Helsinki Declaration.

## Consent

Written and verbal consent with information including the study's details, its purpose, procedures, risks and their rights to withdraw at anytime during the study were obtained from each participant.

## Conflicts of Interest

The authors declare no conflicts of interest.

## Supporting information


**Supplementary Table 1:** Measures of PUA and S‐BN at full bladder (start void), end void and maximum PFM contraction and strain (n = 30) for individual participants.
**Supplementary Table 2:** Change of pubourethral angle (PUA) for individual participants (n = 30).
**Supplementary Table 3:** Change in S‐BN distance for individual participants (n = 30).

## Data Availability

The data that support the findings of this study are available from the corresponding author upon reasonable request.
