# Supplementary material for: Do Ultrasound Measures of Bladder Neck Location and Movement During Pelvic Floor Contraction or Straining Relate to Bladder Neck Support During Voiding?
Source: Neurourol Urodyn. 2026 May 14;45(6):1094–103. doi: 10.1002/nau.70307 (PMC13387904; doi:10.1002/nau.70307)
Supplement: Supplementary file 1 — Supplementary Table 1: Measures of PUA and S‐BN at full bladder (start void), end void and maximum PFM contraction and strain (n = 30) for individual participants. Supplementary Table 2: Change of pubourethral angle (PUA) for individual participants (n = 30). Supplementary Table 3: Change in S‐BN distance for individual participants (n = 30). [file NAU-45-1094-s001.docx]

**Supplementary Table 1: Measures of PUA and S-BN at full bladder (start void), end void and maximum PFM contraction and strain (n=30) for individual participants**

| ID | Parity | PUA at full bladder | PUA at end of void | PUA at max strain | PUA at max PFM contraction | S-BN at full bladder | S-BN at end void | S-BN at max strain | S-BN max PFM contraction |
| --- | --- | --- | --- | --- | --- | --- | --- | --- | --- |
| 1 | Parous | 146.8 | 139.7 | 138 | 121.6 | 28.8 | 27.2 | 22 | 21.6 |
| 2 | Nulliparous | 109.2 | 105.2 | 111.2 | 92.4 | 26.9 | 33.3 | 32.3 | 30.5 |
| 3 | Nulliparous | 151.2 | 155.3 | 161.8 | 104.1 | 29.2 | 28.6 | 28.1 | 24.5 |
| 4 | Parous | 110.1 | 113 | 145.7 | 79.6 | 24.4 | 27.5 | 25.3 | 29.2 |
| 7 | Nulliparous | 125.7 | 142.5 | 130.3 | 86.3 | 23.3 | 22.5 | 26.8 | 24.8 |
| 8 | Nulliparous | 112.7 | 112 | 130.1 | 87.4 | 25.6 | 29.1 | 26.7 | 25.3 |
| 9 | Nulliparous | 105.5 | 106.3 | 124.6 | 90 | 25.8 | 28.7 | 24.1 | 26.1 |
| 10 | Parous | 109.2 | 131.9 | 122.4 | 98.6 | 29.2 | 26.4 | 29.1 | 29.7 |
| 11 | Nulliparous | 119.3 | 135.1 | 139.8 | 92.2 | 23 | 28.3 | 21.5 | 19.5 |
| 12 | Parous | 116.6 | 92.7 | 126.1 | 74 | 27.7 | 31.1 | 26.8 | 25.1 |
| 13 | Nulliparous | 111.4 | 122.2 | 147.2 | 83.5 | 29 | 31.8 | 31.5 | 26.9 |
| 14 | Nulliparous | 107.3 | 120.5 | 141 | 90 | 29 | 30.6 | 29.7 | 29.9 |
| 15 | Nulliparous | 128.3 | 116.3 | 131.2 | 63.5 | 16.6 | 17.4 | 24.3 | 17.3 |
| 16 | Nulliparous | 102.1 | 110.5 | 112.3 | 75 | 21.6 | 26 | 24.4 | 30.5 |
| 17 | Parous | 119.6 | 128 | 127.8 | 90 | 31 | 27.2 | 27.2 | 28.1 |
| 18 | Parous | 154.7 | 138.7 | 150.1 | 108.6 | 23.5 | 19.9 | 21.2 | 17.8 |
| 19 | Parous | 136.6 | 148.4 | 150.8 | 136.7 | 23.5 | 21.6 | 26.5 | 17.7 |
| 20 | Nulliparous | 148 | 148.2 | 169.1 | 101.7 | 32.1 | 31.2 | 32.7 | 26.1 |
| 21 | Nulliparous | 122.1 | 131.4 | 141.8 | 103 | 27.7 | 28.8 | 29 | 34.6 |
| 22 | Parous | 112.6 | 110.5 | 130.3 | 90 | 24.5 | 24.5 | 29.3 | 21.2 |
| 23 | Nulliparous | 130.2 | 137.2 | 135.2 | 94.7 | 25.5 | 25.8 | 28.4 | 26.6 |
| 24 | Nulliparous | 130.8 | 134 | 165.7 | 98.1 | 24.2 | 28.6 | 32.9 | 23.2 |
| 25 | Nulliparous | 127.8 | 137.9 | 143.8 | 69.5 | 24.3 | 24.8 | 24.1 | 26.8 |
| 26 | Nulliparous | 83.9 | 119.4 | 149.3 | 85.7 | 18.9 | 21.5 | 21.3 | 21.2 |
| 27 | Parous | 118 | 100.1 | 146.3 | 80.5 | 30.4 | 34.7 | 33.7 | 34.4 |
| 29 | Parous | 118 | 144.7 | 168.4 | 128 | 29 | 27.5 | 33.6 | 25.6 |
| 30 | Nulliparous | 129.2 | 151.6 | 165.9 | 99.6 | 27.5 | 27.3 | 30.7 | 24.1 |
| 32 | Parous | 120.6 | 121.5 | 124.7 | 84.3 | 26.3 | 25.6 | 28.1 | 26.5 |
| 33 | Nulliparous | 102.3 | 81.2 | 77.6 | 62.5 | 25.1 | 26.9 | 28.1 | 27.3 |
| 34 | Parous | 79.3 | 146.9 | 144.9 | 114.9 | 27 | 21.7 | 24 | 20.9 |

PUA – pubourethral angle (unit of measure – degrees), S-BN – symphysis to bladder neck distance (unit of measure – mm)

Subjects ID no.5 and 28 were not included due to missing data

Subjects ID no. 6 , 31 and 35 were not able to void with the ultrasound transducer on the perineum.

**Supplementary Table 2: Change of pubourethral angle (PUA) for individual participants (n=30)**

| ID | Parity | *Change in PUA – voiding  (end void-full bladder) | Change in PUA – strain  (strain-relax) | Change in PUA – contraction (contract-relax) | **PUA (degrees) maximum mobility |
| --- | --- | --- | --- | --- | --- |
| 1 | Parous | -7.1 | 14.2 | -4.6 | 16.4 |
| 2 | Nulliparous | -4.0 | 14.3 | -7.2 | 18.8 |
| 3 | Nulliparous | 4.1 | 18.3 | -32.9 | 57.7 |
| 4 | Parous | 2.9 | 31.1 | -41.9 | 66.1 |
| 7 | Nulliparous | 16.8 | 6.8 | -38.5 | 44 |
| 8 | Nulliparous | -0.7 | 22.0 | -21.3 | 42.7 |
| 9 | Nulliparous | 0.8 | 25.6 | -10.3 | 34.6 |
| 10 | Parous | 22.7 | 28.1 | -20.5 | 23.8 |
| 11 | Nulliparous | 15.8 | 12.0 | -51.5 | 47.6 |
| 12 | Parous | -23.9 | 26.6 | -25.5 | 52.1 |
| 13 | Nulliparous | 10.8 | 35.1 | -43.4 | 63.7 |
| 14 | Nulliparous | 13.2 | 23.0 | -22.0 | 51 |
| 15 | Nulliparous | -12.0 | 29.1 | -37.7 | 67.7 |
| 16 | Nulliparous | 8.4 | 15.7 | -24.8 | 37.3 |
| 17 | Parous | 8.4 | 14.9 | -26.0 | 37.8 |
| 18 | Parous | -16.0 | 13.8 | -24.1 | 41.5 |
| 19 | Parous | 11.8 | 21.9 | 2.4 | 14.1 |
| 20 | Nulliparous | 0.2 | 22.9 | -61.7 | 67.4 |
| 21 | Nulliparous | 9.3 | 21.2 | -27.7 | 38.8 |
| 22 | Parous | -2.1 | 25.7 | -22.3 | 40.3 |
| 23 | Nulliparous | 7.0 | 15.8 | -30.1 | 40.5 |
| 24 | Nulliparous | 3.2 | 34.8 | -34.1 | 67.6 |
| 25 | Nulliparous | 10.1 | 16.8 | -53.6 | 74.3 |
| 26 | Nulliparous | 35.5 | 41.2 | -29.2 | 63.6 |
| 27 | Parous | -17.9 | 33.1 | -56.6 | 65.8 |
| 29 | Parous | 15.5 | 25.9 | -14.2 | 40.4 |
| 30 | Nulliparous | 31.0 | 54.7 | -40.6 | 66.3 |
| 32 | Parous | 19.2 | 11.0 | -36.0 | 40.4 |
| 33 | Nulliparous | 1.9 | 12.6 | -5.2 | 15.1 |
| 34 | Parous | -2.6 | 24.9 | -22.5 | 30 |

*Voiding movement calculation: end void-full bladder; **Max mobility(PFM) calculation: max strain-max contraction.

Subjects ID no.5 and 28 were not included due to missing data.

Subjects ID no. 6 , 31 and 35 were not able to void with the ultrasound transducer on the perineum

Note that a negative value of PUA relate to elevation of the bladder neck, and a positive value indicate a depression.

**Supplementary Table 3: Change in S-BN distance for individual participants (n=30)**

| ID | Parity | *Change in S-BN – voiding  (end void-full bladder) | Change in S-BN – strain  (strain-relax) | Change in S-BN – contraction (contract-relax) | **S-BN PFM (max mobility) |
| --- | --- | --- | --- | --- | --- |
| 1 | Parous | -1.6 | -3.5 | -3.1 | 0.4 |
| 2 | Nulliparous | 6.4 | -0.5 | -2.5 | 1.8 |
| 3 | Nulliparous | -0.6 | -1.8 | -2.6 | 3.6 |
| 4 | Parous | 3.1 | -2.0 | 6.7 | -3.9 |
| 7 | Nulliparous | -0.8 | -0.1 | -1.9 | -1.3 |
| 8 | Nulliparous | 3.5 | 0.9 | -3.8 | 2 |
| 9 | Nulliparous | 2.9 | -1.0 | -0.1 | 1.4 |
| 10 | Parous | -2.8 | 0.2 | -1.7 | -2 |
| 11 | Nulliparous | 5.3 | 0.4 | -4.4 | -0.6 |
| 12 | Parous | 3.4 | -2.5 | -4.2 | 2 |
| 13 | Nulliparous | 2.8 | 3.1 | -0.7 | 1.7 |
| 14 | Nulliparous | 1.6 | 0.6 | -0.5 | 4.6 |
| 15 | Nulliparous | 0.8 | -1.8 | -9.6 | -0.2 |
| 16 | Nulliparous | 4.4 | 1.0 | 5.6 | 7 |
| 17 | Parous | -3.8 | 0.6 | 0.3 | -6.1 |
| 18 | Parous | -3.6 | 0.4 | -2.2 | -0.9 |
| 19 | Parous | -1.9 | 2.0 | -6.5 | 3.4 |
| 20 | Nulliparous | -0.9 | 1.5 | -8.6 | 8.8 |
| 21 | Nulliparous | 1.1 | -2.8 | 5.3 | 6.6 |
| 22 | Parous | 0.0 | 4.0 | -3.8 | -5.6 |
| 23 | Nulliparous | 0.3 | -0.7 | -2.7 | 8.1 |
| 24 | Nulliparous | 4.4 | 6.3 | -1.4 | 1.8 |
| 25 | Nulliparous | 0.5 | -3.4 | 1.5 | 9.7 |
| 26 | Nulliparous | 2.6 | -2.4 | 3.1 | -2.7 |
| 27 | Parous | 4.3 | 0.4 | 2.8 | 0.1 |
| 29 | Parous | 0.0 | 0.3 | -4.9 | -0.7 |
| 30 | Nulliparous | 1.0 | 4.7 | -3.6 | 8 |
| 32 | Parous | 0.5 | -1.4 | -3.5 | 6.6 |
| 33 | Nulliparous | -0.1 | -4.5 | -2.7 | 1.6 |
| 34 | Parous | -3.0 | 3.6 | -3.2 | 0.8 |

*Voiding movement calculation: end void-full bladder; **Max mobility(PFM) calculation: max strain-max contraction

Subjects ID no.5 and 28 were not included due to missing data.

Subjects ID no. 6 , 31 and 35 were not able to void with the ultrasound transducer on the perineum.

A negative value for S-BN indicate a decrease and a positive value indicate an increase in distance.
